# Case report: Patient specific combination of surgery and immunotherapy in advanced squamous cell carcinoma of the head and neck – a case series and review of literature

**DOI:** 10.3389/fimmu.2022.970823

**Published:** 2022-10-27

**Authors:** Manuel Olmos, Rainer Lutz, Tjark-Ole Büntemeyer, Jacek Glajzer, Christopher-Philipp Nobis, Jutta Ries, Tobias Möst, Markus Eckstein, Markus Hecht, Antoniu-Oreste Gostian, Michael Erdmann, Yannick Foerster, Marco Kesting, Manuel Weber

**Affiliations:** ^1^ Department of Oral and Cranio-Maxillofacial Surgery, Friedrich-Alexander-Universität Erlangen-Nürnberg, Erlangen, Germany; ^2^ Comprehensive Cancer Center Erlangen-European Metropolitan Area of Nürnberg (CCC ER-EMN), Erlangen, Germany; ^3^ Deutsches Zentrum Immuntherapie (DZI), Erlangen, Germany; ^4^ Institute of Pathology, Friedrich-Alexander-Universität Erlangen-Nürnberg, Erlangen, Germany; ^5^ Department of Radiation Oncology, Friedrich-Alexander-Universität Erlangen-Nürnberg, Erlangen, Germany; ^6^ Department of Otorhinolaryngology – Head and Neck Surgery, Friedrich-Alexander-Universität Erlangen-Nürnberg, Erlangen, Germany; ^7^ Department of Dermatology, Friedrich-Alexander-Universität Erlangen-Nürnberg, Erlangen, Germany

**Keywords:** salvage surgery, HNSCC, OSCC, CSCC, immunotherapy, checkpoint inhibition, anti-PD 1, neoadjuvant

## Abstract

**Background:**

Prognosis of patients with recurrent or metastatic head and neck cancer is generally poor. Adjuvant immunotherapy (IT) featuring immune checkpoint inhibition (ICI) is standard of care in advanced stage head and neck squamous cell carcinoma (HNSCC) and cutaneous squamous cell carcinoma (CSCC). ICI response rates in CSCC are described as higher than in HNSCC. IT is constantly shifting into earlier disease stages which confronts the surgeon with immunotherapeutically pre-treated patients. It is therefore becoming increasingly difficult to assess which patients with symptomatic tumor disease and a lack of curative surgical option might benefit from salvage surgery.

**Case presentations:**

The following 6 cases describe therapeutic decision-making regarding ICI and (salvage) surgery in patients with advanced stage HNSCC or CSCC. **Cases A and B** focus on neoadjuvant ICI followed by salvage surgery. In **Cases C and D** salvage surgery was performed after short-term stabilization with partial response to ICI. The last two cases (**Cases E and F**) address the surgical approach after failure of ICI. All cases are discussed in the context of the current study landscape and with focus on individual decision-making. For better understanding, a timetable of the clinical course is given for each case.

**Conclusions:**

ICI is rapidly expanding its frontiers into the neoadjuvant setting, frequently confronting the surgeon with heavily pretreated patients. Salvage surgery is a viable therapeutic concept despite the rise of systemic treatment options. Decision-making on surgical intervention in case of a salvage surgery remains an individual choice. For neoadjuvant ICI monitoring regarding pathological tumor response or tumor necrosis rate, we suggest correlation between the initial biopsy and the definite tumor resectate in order to increase its significance as a surrogate marker. Scheduling of neoadjuvant ICI should be further investigated, as recent studies indicate better outcomes with shorter time frames.

## Background

Incidence rates for head and neck cancer have decreased in recent decades for laryngeal and nasopharyngeal cancer, while they have increased for oral/hypopharyngeal cancer and lip/oral cavity cancer (1). The prognosis of patients with recurrent or metastatic (R/M) HNSCC is generally poor and treatment often associated with high morbidity (2). Primary’s grading, margins and lymph node ratio significantly influence risk of recurrence which occurs in more than half of advanced stage patients (stage III or IV) within 3 years of definitive treatment (2–4). Therefore, the search for innovative treatment options in advanced cases is becoming more and more important. To date, surgical tumor resection and neck dissection combined with radio(chemo)therapy are considered the most effective treatments options for HNSCC in advanced cases (5). In case of locoregional recurrence, salvage surgery or re-irradiation are available (6, 7). The introduction of immunotherapy (IT) featuring immune checkpoint inhibition (ICI) recently provided a further treatment option for advanced solid cancers, including HNSCC (8). As of now, immunotherapy in the recurrent or metastatic (R/M) setting is generally characterized by relatively low response rates of 13.3%, compared with response rates of up to 44% reported for neoadjuvant treatment (9–13). Timing and scheduling of ICI might be crucial for therapy outcome. In this regard, several preclinical and clinical studies indicate that ICI might be more effective at earlier disease states (14–16). Additionally, experimental studies demonstrate that immune responses in the head and neck area are reduced after cervical lymph node dissection (17). For HNSCC neoadjuvant immunotherapeutic treatment is under intense investigation in large clinical trials (NCT03708224, NCT03129061, and NCT03944915). In particular checkmate-141 and KEYNOTE-048 were practice changing trials resulting in the approval of Nivolumab and Pembrolizumab (PD-L1 CPS≥ 1) as first line therapy for patients with R/M HNSCC (9, 18). Completed and published phase II studies describe neoadjuvant use of Pembrolizumab as safe, reporting pathologic response in 44% of patients and a lower 1-year relapse rate compared to historical in patients with high-risk pathology (11, 12). The result of the consecutive phase III study (Keynote-689, NCT03765918) is pending and highly anticipated. Given the current study landscape, a paradigm shift from induction chemotherapy to neoadjuvant ICI may soon occur (19–21).

Cutaneous squamous cell carcinoma (CSCC) is the second most common skin cancer and has seen rising incidence in recent years (22, 23). Many factors are known to increase the risk of CSCC, with cumulative sun exposure being of greatest importance, leading to CSCC developing predominantly in the head-and-neck area (24). In addition, immunosuppression is known to play an important role in the development of CSCC (25, 26). Most cases are cured by complete surgical excision (27, 28). However, a substantial number of patients subsequently develop either metastatic or locally advanced CSCC not amenable to curative surgery or curative radiotherapy. For better understanding, these cases are referred to as advanced CSCC in the following. According to the National Comprehensive Cancer Network (NCCN) Guidelines V2.2021, systemic ICI with Cemiplimab or Pembrolizumab is recommended only for complicated cases of advanced CSCC when curative radiotherapy or surgery is not available (28). Regarding neoadjuvant ICI for CSCC of the head and neck, recently published Phase II trial results report that administration is safe. Complete pathologic response (cPR) was observed in 55% of the patients with additional 15% of patients showing major pathologic response (MPR, 10% viable tumor) (29, 30). Interestingly, response rates are described as higher compared to ICI in HNSCC (31–33).

In highly advanced or recurrent cases of both HNSCC and CSCC and at the transition from the curative to the R/M setting salvage surgery can be considered as an alternative or addition to re-irradiation in selected cases (34, 35). With an increasing number of long-term responders on immunotherapy in the R/M setting there is a growing number of patients with tumor-derived symptoms and quest for surgical intervention. In addition, ICI is constantly shifting to earlier stages of disease, confronting the surgeon with immunotherapeutically pre-treated patients. In the context of this development, it is becoming increasingly difficult to assess which patients with symptomatic tumor disease and no curative surgical option could benefit from salvage surgery.

Individual decision-making for surgical therapy in the context of IT using ICI will be discussed on the basis of the following 6 cases. In addition, we will provide a review of the current literature in order to facilitate future therapeutic decisions for surgeons.

## Materials and methods

PubMed and Scopus were comprehensively searched in January 2022 using the keywords *HNSCC*, *cSCC*, *immunotherapy* and *salvage surgery*. The individual keywords *HNSCC* and *cSCC* were combined with *immunotherapy* and *salvage surgery*. It was then updated in February 2022 and limited to a publication date within the last 15 years. The search returned 18 results on PubMed, 6 on Scopus. Subsequently, the results were supplemented by a manual search. For better comprehension, a table of current human studies on neoadjuvant immunotherapy of HNSCC is attached (**Table 1**).

**Table 1:** Human studies on neoadjuvant Immunotherapy in HNSCC.

Year	Authors	Title	Study design	Patient dollective	Neoadjuvant intervention	Target checkpoint	Surgical intervention	Adjuvant therapy	Primary outcome measures and results
2022	Wise-Draper T. M., et al. (36)	Phase II Clinical Trial of Neoadjuvant and Adjuvant Pembrolizumab in Resectable Local-Regionally Advanced Head and Neck Squamous Cell Carcinoma	multicenter, nonrandomized two arm phase II trial	92 patients, locally advanced HNSCC, resectable, clinical stage III–IV (oral cavity, larynx, hypopharynx or p16-negative oropharynx)	Neoadjuvant pembrolizumab (200 mg) 1 to 3 weeks prior to surgery	anti–PD-1	Gross total surgical resection, not further specified	Adjuvant radiotherapy + adjuvant pembrolizumab, additional chemotherapy in patients with positive margins and/or extranodal extension	Pathological response in 39% of the patients (defined as tumor necrosis with associated histiocytic inflammation and/or giant cell reaction to keratinaceous debris as a percentage of overall tumor bed)
2021	Vos J.L., et al. (13)	Neoadjuvant immunotherapy with nivolumab and ipilimumab induces major pathological responses in patients with head and neck squamous cell carcinoma	nonrandomized two arm phase ll trial	33 patients (26 COMBO and 6 MONO ICI, locoregionally advanced HNSCC, resectable, clinical stage ll-lV, N0−N3b, M0 primary or recurrent (oral cavity, larynx, hypopharynx or oropharynx)	Neoadjuvant nivolumab (240 mg, weeks 1 and 3) or nivolumab + ipilimumab (240 mg and 1 mg kg−1, week 1) followed by nivolumab (week 2) prior to surgery in week 5−6.	anti-PD-1 and anti-CTLA-4	Tumor resection, therapeutic/prophylactic neck dissection and pedicled or free-flap reconstruction	With or without adjuvant radio(chemo)therapy	Pathological response in 35% of the patients after COMBO ICI (arm 2) and 17% after MONO ICI (arm 1) (defined as the %-change in primary tumor viable tumor cell percentage from baseline biopsy to on-treatment resection)
2021	Ferrarotto R, et al. (30)	Pilot Phase II Trial of Neoadjuvant Immunotherapy in Locoregionally Advanced, Resectable Cutaneous Squamous Cell Carcinoma of the Head and Neck	singlecenter, pilot phase ll trial	20 patients, newly diagnosed or recurrent stage III–IVA CSCC-HN patients amenable to curative-intent surgery	Neoadjuvant cemiplimab (two cycles, 350 mg) every 3 weeks before surgery; surgery ≥21 days after the second cemiplimab dose	anti–PD-1	Oncologic surgical resection according to the original clinical and radiologic extent of disease	Adjuvant radiotherapy planned at baseline for all patients; adjuvant therapies were reconsidered by the multidisciplinary team on a case-by-case basis after surgery du to impressive pathologic responses	Pathologic response in 75% of the patients with complete response in 55% (defined as the absence of viable tumor in the posttreatment surgical specimens) and major response in 20% of cases (defined as ≤10% viable tumor)
2021	Hanna G.J., et al. (10)	Neoadjuvant and Adjuvant Nivolumab and Lirilumab in Patients with Recurrent, Resectable Squamous Cell Carcinoma of the Head and Neck	multicenter, nonrandomized single arm phase II trial	28 patients, locoregionally recurrent HNSCC (oral cavity, oropharynx, larynx or hypopharynx) with disease free interval >8 weeks after completion of prior therapy	Neoadjuvant nivolumab (240 mg) plus neoadjuvant lirilumab (240 mg) 1 to 3 weeks prior to surgery	anti–PD-1 and anti-KIR	Surgical procedure and neck management at the discretion of the treating head and neck surgeon(s) based on pre-treatment clinical and radiologic assessment	Adjuvant immunotherapy (six cycles of adjuvant nivolumab and lirilumab)	Pathological response in 43% of the patients with major response in 14% (defined as tumor viability, TV ≤ 10%) and partial response in 29% (defined as TV≤ 50%).
2021	Leidner R., et al. (37)	Neoadjuvant immunoradiotherapy results in high rate of complete pathological response and clinical to pathological downstaging in locally advanced head and neck squamous cell carcinoma	singlecenter, four-arm phase 1b trial	21 patients, previously untreated locally advanced p16- positive (stages I–III) and p16- negative (stages III– IVA) HNSCC	Neoadjuvant nivolumab (three cycles, 240 mg) plus neoadjuvant radiotherapy (either 40 Gy in 5 fractions or 24 Gy in 3 fractions)	anti–PD-1	Surgery in all cohorts was planned 5 weeks post radiotherapy	Adjuvant immunotherapy (nivolumab 480 mg intravenous q 4 weeks for 3 doses starting 4 weeks after surgery)	Major pathological response and pathological complete response rates were 86% and 67% (cPR was defined as the absence of viable tumor cells, mPR was defined as fewer than 10% viable tumor cells)
2021	Oliva M., et al. (14)	Antitumor immune effects of preoperative sitravatinib and nivolumab in oral cavity cancer: SNOW window-of-opportunity study	singlecenter, nonrandomized single arm trial	10 patients, newly- diagnosed untreated T2- 4a, N0- 2 or T1 >1 cm- N2 oral cavity carcinomas	Neoadjuvant sitravatinib (120 mg) daily from day 1 up to 48 hours pre- surgery and one dose of nivolumab (240 mg) on day 15 (surgery between day 23 and 30)	anti–PD-1 and tyrosine kinase inhibitor	Tumor resection, ipsilateral (and contralateral, in some patients) therapeutic/prophylactic neck dissection and reconstruction	Adjuvant radiotherapy or radio(chemo)therapy as per standard of care	One case of complete pathological response and two cases of major pathological response (cPR was defined as the absence of viable tumor cells, mPR was defined as fewer than 10% viable tumor cells)
2020	Uppaluri R, et al. (11)	Neoadjuvant and Adjuvant Pembrolizumab in Resectable Locally Advanced, Human Papillomavirus-Unrelated Head and Neck Cancer: A Multicenter, Phase II Trial	multicenter, nonrandomized single arm phase II trial	36 patients, locally advanced HNSCC, resectable, clinical stage III–IVb (oral cavity, larynx, hypopharynx or p16-negative oropharynx)	Neoadjuvant pembrolizumab (200 mg) 2 to 3 weeks prior to surgery	anti–PD-1	Tumor resection, therapeutic/prophylactic neck dissection and pedicled or free-flap reconstruction	Adjuvant radio(chemo)therapy + adjuvant pembrolizumab in patients with positive margins and/or extranodal extension	Pathological response in 44% of the patients (defined as tumor cell necrosis and keratinous debris as a percentage of overall tumor bed); 1-year relapse rate 16.7% for high-risk pathology
2020	Schoenfeld J.D., et al. (38)	Neoadjuvant Nivolumab or Nivolumab Plus Ipilimumab in Untreated Oral Cavity Squamous Cell Carcinoma A Phase 2 Open-Label Randomized Clinical Trial	singlecenter, randomized two arm phase ll trial	29 patients, previously untreated squamous cell carcinoma of the oral cavity (≥T2, or clinically node positive)	Neoadjuvant nivolumab (3mg/kg, weeks 1 and 3) or nivolumab and ipilimumab (1 mg/kg, week 1 only); surgery 3 to 7 days after the second cycle	anti-PD-1 and anti-CTLA-4	Gross total surgical resection, not further specified	With or without adjuvant radio(chemo)therapy	Pathological response was assessed as pathologic tumor response 0-2; PTR1: 38%with nivolumab, 40% with nivolumab and ipilimumab; PTR2: 15%with nivolumab, 33% with nivolumab and ipilimumab (defined as PTR0 = no or <10% response, PTR1 = ≥10% and <50%, and PTR2 = ≥50%)

Recent human studies on neoadjuvant immunotherapy in HNSCC including information on year of publication, authors, title, study design, patient collective, neoadjuvant intervention, target checkpoint, surgical intervention, adjuvant therapy as well as primary outcome measures and result.

## Case presentations

The following two cases describe successful combination of neoadjuvant ICI and salvage surgery:

### Case A

**Figure 1:**
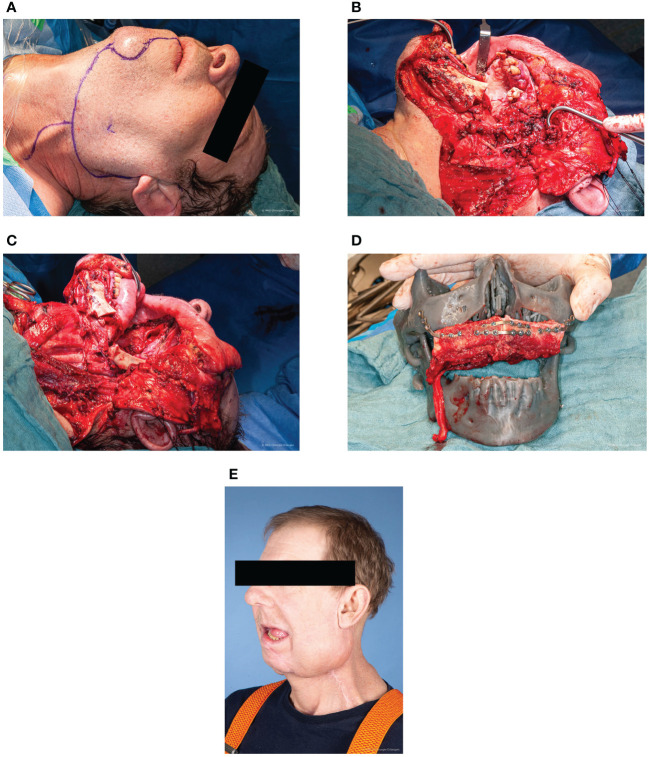
Case A **(A)** Preoperative sketching of the surgical incision. Photo taken under intubation anaesthesia. Preauricular and submandibular approach with temporary lip-split mandibulotomy. **(B)** Intraoperative view of the tumor on the left maxilla. Situs after successful access *via* preauricular and submandibular with temporary lip-split mandibulotomy with view of the tumor in the maxilla. **(C)** Intraoperative view after bilateral neck dissection and radical tumor resection. Neck dissection level I-V left and I-III right with vascular presentation on both sides. Radical tumor resection by way of bilateral maxillectomy. **(D)** CAD-CAM assisted microvascular double flap reconstruction. In domo planned and printed surgical model for sagittal positioning of the microvascular 2-segment fibula transplant. An additional microvascular latissimus dorsi graft was used for enoral soft tissue reconstruction. **(E)** Last follow-up in early-April 2022 - 5 months postoperative. No signs of tumor recurrence. Functionally satisfying rehabilitation with good mouth opening.

**Figure 2:**
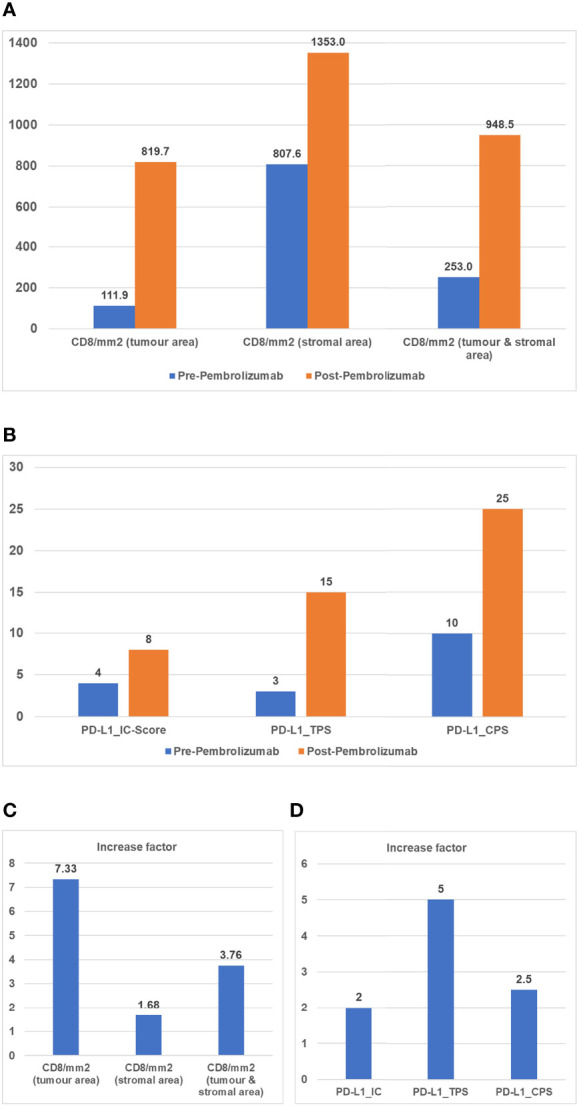
**(A)** CD8 density (mm2) (Cytotoxic T cell marker) during treatment. Points of interest/sample collection: initial biopsy (blue); final resection after Pembrolizumab (orange). Charts show: CD8+ cells per mm2 in tumor area; CD8+ cells per mm2 in stroma area; CD8 mm2 combined tumor and stroma area. **(B)** Immune scores during treatment. Points of interest/sample collection: initial biopsy (blue); final resection after Pembrolizumab (orange). Charts show: TC, tumor cells; TPS, tumor proportion score (stained tumor cells/tumor cells); IC-Score, inflammatory cell score (stained inflammatory cells/tumor surface); CPS, combined positivity score [(stained tumor cells + stained mononuclear immune cells)/tumor cells]. **(C)** Increase factor in CD8 density (mm2). Increase factor between initial biopsy and final resection in terms of CD8/mm2 tumor area, CD8/mm2 stromal area and CD8/mm2 tumor and stromal area. **(D)** Increase factor in Immune scores. Increase factor between initial biopsy and final resection in terms of immune scores. IC-Score, inflammatory cell score (stained inflammatory cells/tumor surface); TPS, tumor proportion score (stained tumor cells/tumor cells); CPS, combined positivity score [(stained tumor cells + stained mononuclear immune cells)/tumor cells].

Patient A presented at the end of October 2021 with pain in the right maxilla and restricted mouth opening. Subsequent computed tomography (CT) and biopsy confirmed the diagnosis OSCC of the left maxilla (cT4b cN1; HPV/p16 negative). Immunohistochemical staining of the biopsy revealed a tumor proportion score (TPS, stained tumor cells/tumor cells) of 15%, inflammatory cell score (IC-Score, stained inflammatory cells/tumor surface) of 0,5% and a combined positive score (CPS, (stained tumor cells + stained mononuclear immune cells)/tumor cells)) of 15. A single dose of Pembrolizumab 200 mg was administered on October 2021, 15 days prior to surgery. Operation included radical tumor resection with lip-split mandibulotomy as demonstrated in **Figures 1A, B**, and bilateral selective neck dissection level I-V left and I-III right (**Figure 1C**). CAD-CAM assisted (**Figure 1D**) microvascular reconstruction was used for defect coverage. Pathological assessment revealed: ypT4a pN1 (1/63) L0 V0 Pn0with positive margins to the medial maxillary sinus wall (70% vital tumor cells, 30% regressive change; pathologic tumor response (pTR) can therefore be classified as pTR-1 (10%-49%) (11)). Subsequently, immunohistochemical assessment of the tumor mass was performed. Methods were identical to our previously published case report on neoadjuvant IT (39). A significant factor increase was observed in TPS, IC-Score, CPS and immune cell infiltration (CD8/mm2), comparing initial biopsy and final tumor resection (**Figures 2A–D**). Adjuvant RCT was recommended by the tumor board starting in February 2022. Last follow-up took place in early-April 2022 and showed no signs of tumor recurrence (**Figure 1E**).

### Case B

Patient B presented with OSCC of the right mandible in early January 2021. In detail, histopathological examination resulted in focally moderately differentiated squamous cell carcinoma with extensive necrosis. Staging CT revealed multiple ipsilateral lymph node metastases at level 1-5 (40), partial contact (> 180°) with the internal carotid artery at the skull base and metastasis in the right upper lobe of the lung; cT4a N3b M1 (HPV/p16 negative). Neoadjuvant ICI (TPS 100%, IC 0.5%, CPS 100) was administered in mid-January with Pembrolizumab 200 mg, 17 days prior to surgery. Preoperative restaging after neoadjuvant therapy revealed local tumor progression and predominantly size-progressive necrotic lymph nodes in level I-IV on the right. Surgery took place at the beginning of February including partial mandibulectomy, bilateral level I-V neck dissection and microvascular flap reconstruction. Histologic assessment confirmed clear margin resection for OSCC ypT4a pN3b (7/41) L0 V0 Pn0 [40% vital tumor cells, 60% regressive change; pTR-2 (≥50%) (11)]. Postoperative CT to clarify an atypical awakening process showed multifocal cerebral infarcts with proximal complete occlusion of the right external carotid artery the patient was transferred to the intensive care unit of the neurology department for early neurological rehabilitation. After significant improvement of the general condition, adjuvant radiotherapy (64 Gy total dose) was initiated in June 2021. The last follow-up took place in late-February 2022 and showed no signs of tumor recurrence.

The occurrence of peri-/postoperative cerebrovascular apoplexy following neoadjuvant ICI in **Case B** is not considered a side effect of IT, but a general complication due to the length and complexity of surgery.

The following two cases describe the combined administration and feasibility of ICI and salvage surgery in the R/M setting. Surgery was performed after partial response to ICI or delay of tumor progression:

### Case C

**Figure 3:**
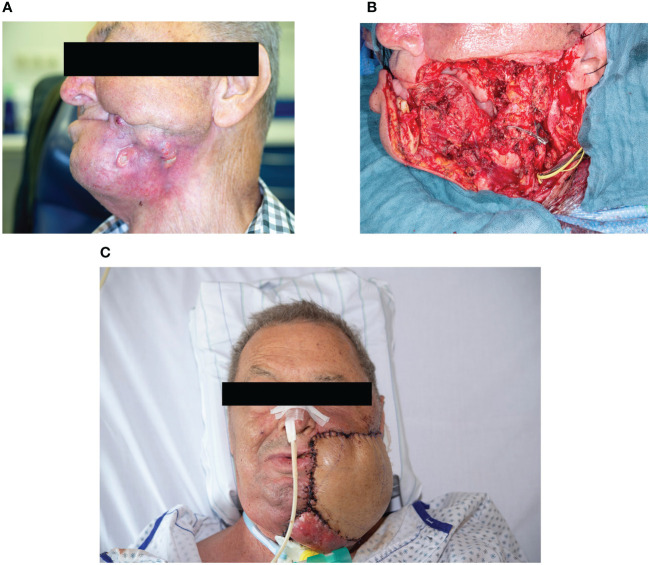
Case C **(A)** Abscess and multiple skin fistulas on the left paramandibular side due to cutaneous metastasis under IT. Photo taken during tumor aftercare from the left-hand side. **(B)** Salvage surgery. Tumor resection with hemimandibulectomy of the left side and partial resection of the left buccal region/maxilla. **(C)** Reconstruction with two microvascular grafts. Microvascular double flap reconstruction with anterior lateral thigh and latissimus dorsi flap.

The patient was initially treated with a squamous cell carcinoma of the left oropharynx in mid-2014 by primary RCT (72 Gy total dose; cisplatin and 5-FU). Mid-2017, OSCC of the left buccal area was diagnosed. It was treated surgically by resection and microvascular flap reconstruction as well as bilateral neck dissection due to radiologically enlarged cervical lymph nodes (pT1 pN0 G2 R0; HPV/p16 negative). Adjuvant therapy was not carried out. In late-2018, the patient was diagnosed with bladder cancer which was treated by primary surgical resection (pT2 G3) and adjuvant RCT (59 Gy total dose; carboplatin and 5-FU). Shortly afterwards, biopsy of the left maxillary premolar area and buccal mucosa revealed recurrence of OSCC (rcT4 rcN2b cM0 G2). The patient was treated by definitive RCT (66 Gy and carboplatin+5-FU). Due to overall poor prognosis of re-irradiation in advanced OSCC/HNSCC this was followed by four months of ICI with Pembrolizumab 200mg (TPS 2%, IC 3%, CPS 5). After four months and initial improvement of the clinical condition, systemic therapy was switched from Pembrolizumab to Paclitaxel and Cetuximab due to tumor progression. The patient’s clinical condition progressively deteriorated, with abscess and multiple skin fistulas on the left paramandibular side due to cutaneous metastasis (**Figure 3A**). In order to improve the clinical condition, the decision was made to carry out salvage surgery. Tumor resection with hemimandibulectomy and partial resection of the left buccal region/maxilla was performed (**Figure 3B**) followed by microvascular flap reconstruction (**Figure 3C**). Histology revealed a tumor mass of approximately 6.5 cm poorly differentiated OSCC with bone infiltration (rpT4a L0 V0 Pn0 G3 R0). Close margin resection with minimal 0.2 cm margins was achieved. Despite close margin resection, CT in January 2021 showed early local recurrence and multiple predominantly new bi-pulmonary consolidations. The patient died of tumor-toxic multi-organ failure in April 2021.

### Case D

**Figure 4:**
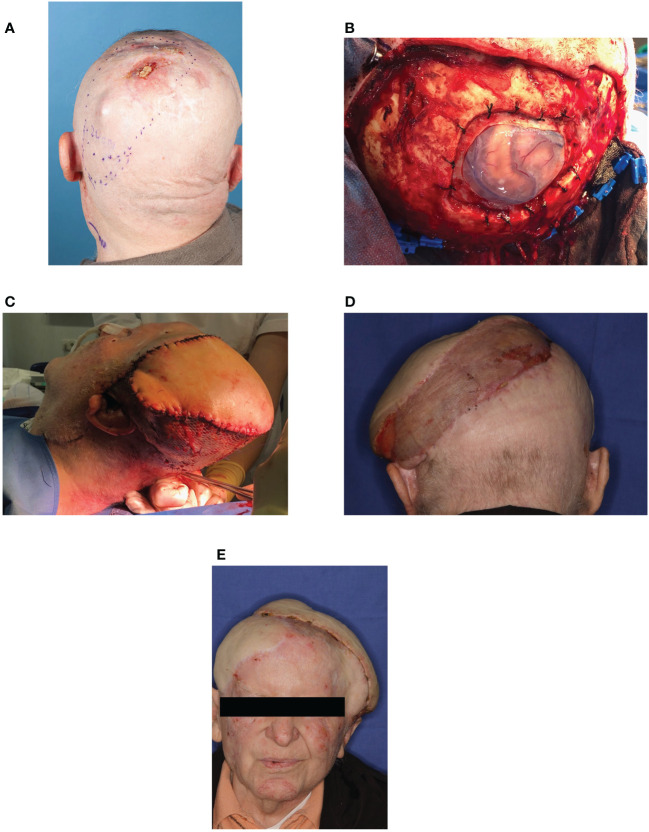
Case D **(A)** Recurrence of the outer skin with in-transit metastasis. Extensive recurrence of the outer skin located occipital-left and measuring 7 cm with arrosion of the skull calvaria and the dura mater as well as an in-transit metastasis. **(B)** Salvage surgery. Salvage surgery with palliative intent by radical tumor resection including resection of the calvarium and dura mater. **(C)** Reconstruction with microvascular graft. Defect coverage with a microvascular latissimus dorsi graft in collaboration with the Department of Neurosurgery. **(D)** Last follow-up in August-2021 - 2 months postoperative. No signs of tumor recurrence. Further follow-up care takes place near home at the patient’s request. **(E)** For further information see **Figure 4D**.

After resection and radiotherapy of multiple CSCC of the head and neck region from 2008 to 2019, Patient D presented with field changes of the scalp in Mai 2019. In August 2019, ICI was initiated with palliative intent by administration of 2 doses of Cemiplimab by the Department of Dermatology. In the course, regression of all lesions was observed except for one parietal and one lateral-cervical lesion. The interdisciplinary tumor board recommended salvage surgery to treat the persistent lesions. Salvage surgery took place in September 2019 by radical tumor resection and microvascular flap reconstruction. Subsequent histology revealed ulcerated, poorly differentiated cutaneous squamous cell carcinoma (cervical R0, parietal R1). The postoperative result was found to be aesthetically and functionally good. Due to progression of lesions of other localization and R1 resection of the parietal lesion, experimental use of intralesional mRNA TLR agonist was decided as part of the CureVac trial (cohort A, NCT03291002). The administration took place from November 2019 to February 2020. After discontinuation of IT due to further progress, an extensive recurrence of the outer skin occipital-left measuring 7 cm with arrosion of the skull calvaria and the dura mater as well as an in-transit metastasis was diagnosed in May 2021 **(** **Figure 4A** **)**. Salvage surgery with was performed in June 2021 by radical tumor resection including resection of the calvarium and dura mater and defect coverage with a microvascular flap in collaboration with the Department of Neurosurgery (rpT3 rpN0 L0 V0 G3 Pn0) **(** **Figures 4B, C** **)**. Last CT and tumor aftercare in August 2021 showed no signs of recurrence (**Figures 4D, E** **)**. Further aftercare takes place close to home according to patient’s choice.

The following two cases describe the implementation of salvage surgery after failure of (chemo/radio-) immunotherapy:

### Case E

Patient E presented in mid-May 2017 with OSCC of the left floor of the mouth. In late May 2017, primary surgical therapy was performed by means of partial mandibulectomy, floor of the mouth reconstruction and level I-III right and I-V left neck dissection (pT2 pN0 (0/37) L0 V0 Pn0 cM0 with close margins; HPV/p16 negative). Re-resection due to close margins was performed shortly after. Postoperative interstitial brachytherapy with 50 Gy total dose was administered. In early March 2018, CT scan revealed urgent suspicion of a recurrence with pharyngeal location close to the ramus mandibulae. Biopsy showed a poorly differentiated basaloid squamous cell carcinoma (G3). The interdisciplinary tumor board decided on a primary RCT starting at the end of June with a total dose of 50.4 Gy and 5-FU as well as cisplatin due to possible infiltration of the prevertebral fascia. Staging-CT 4 months after RCT initiation showed size regressive oropharyngeal carcinoma however multiple new lymph node and lung metastases which were subsequently treated by stereotactic radiation with 36 Gy total dose. Due to the generally poor prognosis with re-irradiation, additional IT with Pembrolizumab was initiated in November 2018. Based on a significant deterioration of the clinical condition with strong growth of submental and submandibular metastases under IT, palliative resection of metastases and microvascular flap reconstruction was performed in February 2019. Immunotherapy was continued. The patient died 4 months later with unknown cause of death.

### Case F

In April 2019 Patient F presented with G3 OSCC of the tongue base (cT4a cN3b cM0; HPV/p16 negative). Induction chemo-immunotherapy was initiated as part of the CheckRad-CD8 study (NCT03426657) (41); for exact schedule see **Table 2f**. Four weeks after completion of therapy, control FDG-PET/CT revealed a tongue base carcinoma that had progressed in size and metabolism under induction chemo-immunotherapy, as well as progressive lymph node metastases. Based on tumor progression, tumor board decided on a salvage surgical procedure. At the end of June, partial mandibulectomy, bilateral neck dissection level I-V and subsequent reconstruction was carried out by an interdisciplinary team of ENT and maxillofacial surgeons (ypT4a ypN3b (2/77) cM0 L1 V0 Pn1 with close margins). In addition, and due to close margin resection, adjuvant RCT with 60 Gy total dose and cisplatin/5-FU was administered from early August to early November 2019. The follow-up CT at the beginning of December showed no local recurrence of the floor of the mouth, but three pulmonary foci of progressive size. After close follow-up, it was decided in mid-February 2020 to repeat chemotherapy according to the TPExtreme regimen (**Table 2f**) due to strong progression of pulmonary metastasis. Further progression of lung lesions with cavern formation was diagnosed in February 2021. Supportive-palliative procedure was recommended and carried out near home.

**Table 2:** Time history.

Date	Intervention/Event
**2a**
10/2021	**OSCC** left maxilla cT4b cN1; TPS 15%, IC 0,5%, CPS 15
10/2021	Immunotherapy: **Pembrolizumab 200 mg**
11/2021	Surgical resection pT4a pN1 (1/63) L0 V0 Pn0 R1; bilateral neck dissection, reconstruction by fibula and latissimus-dorsi graft; immunohistochemical staining
02/2022	Adjuvant RCT
	Demographics: Male born 12/1962 Age at start of therapy: 53
**2b**	
01/2021	**OSCC** right mandible cT4a N3b M1; TPS 100%, IC 0.5%, CPS 100 CT: multiple ipsilateral lymph node metastases; partial contact to the internal carotid artery
01/2021	Immunotherapy: **Pembrolizumab 200mg**
01/2021	Restaging CT: tumor progression with new midline tumor infiltration; predominantly size-progressive necrotic lymph nodes on the right
02/2021	**OSCC** ypT4a pN3b (7/41) L0 V0 Pn0 (40% vital tumor cells, 60% regressive change; TR-2); surgical resection, neck dissection level I-IV on the right and reconstruction by latissimus dorsi graft and an osteosynthesis plate
02/2021	Patient transferred to ICU due to atypical awakening process; revision of the latissimus-dorsi-graft due to venous congestion
06/2021	Adjuvant radiotherapy (64 Gy total dose)
01-02/2022	Last follow-up and CT without evidence of local recurrence and or lymph node metastases
	Demographics: Male born 12/1964 Age at start of therapy: 56
**2c**	
2010-2014	History of multiple CIS treated surgically
06/2014	**HNSCC** left oropharynx cT1 cN0 cM0 G2; primary RCT (72 Gy total dose and cisplatin with 5-FU)
09/2017	**OSCC** left buccal region pT1 pN0 G2 R0; surgical resection, bilateral neck dissection and reconstruction by radial forearm flap
11/2018	Bladder carcinoma pT2 cN0 cM0 G3; surgical resection and RCT (59,4 Gy total dose with Cisplatin and 5-FU)
01/2019	Recurrence of **OSCC** buccal region rcT4 rcN2b cM0 G2 (**PD-L1-positive**); definitive RCT (66,6 Gy total dose and cisplatin with 5-FU);
07/2019	CT: slightly regressive OSCC
08/2019 – 02/2020	Immunotherapy: **Pembrolizumab** as maintenance treatment after re-irradiation
since 02/2020	Systemic therapy changed to **Paclitaxel** and **Cetuximab** due to tumor progression
09/2020	**Salvage surgery** due to progressively deteriorating clinical conditions
01/2021	Early recurrence of OSCC with multiple bipulmonary consolidations
04/2021	Death under BSC
	Demographics: Male born 11/1942 Age at start of (in domo) therapy: 71
**2d**	
2008-2019	multiple **cSCC** of the head and neck region; resection and radiotherapy
08/2019	Immunotherapy: **Cemiplimab**
09/2019	**cSCC** of the scalp and neck; **salvage surgery (1)** (cervical R0, parietal R1), reconstruction by latissimus dorsi graft and local rotational flap
11/2019-02/2020	mRNA TLR agonist: CureVac trial (cohort A, NCT03291002)
07/2020	**cSCC** parietal; surgical resection, reconstruction by split skin graft
06/2021	**cSCC** extensive recurrence occipital left; arrosion of the skull calvaria and the dura mater; in-transit metastasis; **salvage surgery (2)** rpT3 rpN0 L0 V0 G3 Pn0 R0, reconstruction by latissimus dorsi graft
07/2021	Wound revisions and revision of microanastomosis with venous interposition due to malperfusion
08/2021	CT: no indication of recurrence; further aftercare takes place close to home
	Demographics: Male born 11/1947 Age at start of (in domo) therapy: 71
**2e**	
05/2017	**OSCC** pT2 pN0 (0/37) L0 V0 Pn0 cM0; surgical resection; neck dissection; reconstruction by microvascular radial forearm flap
09/2017	RT; brachytherapy with 50 Gy total dose
03/2018	**OSCC Recurrence** parapharyngeal
06-08/2018	Primary RCT; 50.4 Gy total dose; 5-FU and cisplatin
10/2018	CT: size-regressed oropharyngeal carcinoma under RCT; multiple lymph node and lung metastases
11/2018-06/2019	Immunotherapy: **Pembrolizumab 200 mg** (q21d)
02/2019	**Salvage surgery** due to progressively deteriorating clinical conditions; resection of the submandibular metastasis, R0 resection, reconstruction by microvascular anterior-lateral thigh graft
06/2019	Death with unknown cause
	Demographics: Male born 10/1963 Age at start of therapy: 53
**2f**	
04-05/2019	**OSCC** cT4a cN3b cM0; IC (CheckRad-CD8; NCT03426657): Docetaxel (75 mg/m2 body surface), day 1-3 Cisplatin (30 mg/m2 body surface), day 1-3 Tremelimumab (75 mg, fix dose), day 5 Durvalumab (1500 mg, fix dose), day 5
05/2019	CT: tumor and metastases progression under immuno-chemotherapy
06/2019	**OSCC** ypT4a ypN3b (2/77) cM0 L1 V0 Pn1; local R1); **salvage surgery**, bilateral neck dissection (I-V), reconstruction by latissimus dorsi flap
08-11/2019	RCT with 60 Gy total dose and cisplatin/5-FU
12/2019	CT: no local recurrence of the floor of the mouth; three pulmonary round foci of progressive size
02/2020	Chemotherapy TPExtreme regimen: Cetuximab (400mg loading dose; maintenance dose two times with 200 mg) Docetaxel (75 mg/m2 body surface) Carboplatin AUC 5 q3w
02/2021	Tumorboard decision: supportive-palliative procedure with no interventional procedure
	Demographics: Male born 11/1980 Age at start of therapy: 38

Brief summary of the clinical course. Patient demographics.

Apart from those described, no ADRs occurred over the course of the cases described.

## Discussion and conclusion

### IT scheduling and monitoring

Cases A and B, as well as our previously published case report on neoadjuvant immunotherapy (39), show that neoadjuvant ICI can be performed successfully and should be evaluated in selected cases. Nevertheless, the optimal timing for neoadjuvant ICI remains unclear. Uppaluri et al. recommend a time frame of 13-22 days prior to surgery (11). In the ongoing and highly anticipated Keynote-689 trial, Pembrolizumab 200 mg is administered on day 1 of a 21-day cycle for 2 cycles (NCT03765918). Regarding our cases, Pembrolizumab 200 mg was given 15 (**Case A**), 17 (**Case B**) and 19 days (39) before surgery. – Reason was not to delay the planned surgery and to stick to the seemingly biologically reasonable time frame given in the literature available at the time (11). Recently, Liu et al. investigated the sole timing of ICI in relation to resection of the primary tumor and its impact on the efficacy of ICI in an animal study. First step was to demonstrate the significantly greater therapeutic efficacy of neoadjuvant compared to adjuvant ICI to eradicate distant metastases following primary tumor resection of breast cancer. Different metastatic burden at the time of treatment was eliminated as a factor by varying the schedule of neoadjuvant and adjuvant ICI administration. Furthermore, investigation on the optimal scheduling between neoadjuvant immunotherapy and surgery showed that short duration (4-5 days instead of 10 days) between first administration of neoadjuvant ICI and resection of the primary tumor yielded in optimal results in overall and relapse-free survival. Interestingly, adding 4 adjuvant doses had no benefit on overall survival and instead lead to an increase in immune related adverse events (15, 16). Comparing the studies described in **Table 1** regarding the publication date and the administration period, it can be observed that more recent studies, e.g. Wise-Draper et al. (36) work according to a shorter administration schedule as described by Liu et al. (16).

Although timing of neoadjuvant ICI differed significantly from the optimal administration schedule proposed by Liu et al. (16), all cases presented, as well as the previously published case, can be classified as pTR-2 9 [**Cases B and** (39)] or pTR-1 (**Case A**) according to the histologic response classification proposed by Uppaluri et al. (11). PTR ≥1 correlates with better clinical outcome, as shown by the aforementioned author (11).

To date, all studies on neoadjuvant ICI in OSCC/HNSCC consider pathological tumor response as an important target criterion for response to therapy. How reliable and feasible is this surrogate marker in the context of other available outcome criteria such as overall survival, immune cell infiltration and radiological response?

In a recently published review, Menzies et al. report high pathological and radiological response rates with impressive recurrence-free survival (RFS) and OS with neoadjuvant therapy in melanoma. The degree of pTR was found to strongly correlate with RFS and OS, allowing the early endpoint to be used as a surrogate marker for survival in future clinical trials (42). This statement is in line with Uppaluri et al. who suggest pTR as a biomarker for response to neoadjuvant ICI and a lower rate of disease relapse (11). The hypothesis is currently being tested in an ongoing phase III trial (NCT03765918). Additionally, pTR seems to be associated with tumor PD-L1 expression and high disease-free survival (DFS) in intermediate-risk patients as stated by Wise-Draper et al. (36, 43). Although Menzies et al. report a correlation between radiological and pathological response, radiological response seems to be inconsistent and unsuitable as a marker for neoadjuvant therapy due to the short time frame and the possibility of radiologic flare as stated by Leidner et al. (37, 42, 44). A recent study by Vos et al. proposes [18F] FDG-PET for pathological response assessment in early neoadjuvant ICI, however radiological flare is also present in PET-CT (45). Regardless, it should be considered that ICI’s effect on the primary tumor site and on potential lymph node metastases may differ (46). Besides radiological and pathological response, the role of immune biomarkers and circulating tumor DNA (ctDNA) seems to be emerging for the assessment of (neoadjuvant) ICI. PD-L1 immune cell area and intratumoral CD8+ cell density have previously been identified as significant positive predictors of pathologic complete response (pCR) (41, 47). Interestingly, the immunohistochemical assessment of **Case A** showed a strong increase in all immunohistochemical parameters examined, including immune cell infiltration (**Figures 4A–C**), despite showing lowest pTR of the three described cases of neoadjuvant ICI. For ctDNA, DNA released from damaged or apoptotic tumor cells, correlation with increased risk of recurrence has been described for several other malignancies (48, 49). Due to its high sensitivity for tumor recurrence (50), it could play a role in the future in HNSCC/OSCC, both for estimating the molecular tumor burden and for progression monitoring. Another promising approach presented by Rozeman et al. shows that both tumor mutation burden and a high interferon-gamma-related gene expression score (IFN-Y score) are associated with a high pathological response and a low risk of relapse in melanomas treated with ipilimumab plus nivolumab. A recent review also mentions tumor mutational burden and IFN-Y score as promising prognostic and predictive markers, highlighting the tumor microenvironment as a potential source for identifying new biomarkers (51, 52).

Currently, pTR/pCR seems to be the most reliable and practical surrogate marker for neoadjuvant ICI response available. However, the significance of pTR/pCR with regard to the necrosis rate of the initial biopsy has received little to no attention in previous publications. As demonstrated in Case B, initial biopsy taken ahead of neoadjuvant therapy may already show extensive necrosis and thus relativize the significance of any pTR/pCR determined on the basis of the definite tumor resectate. As a solution, we propose to relate the pTR/pCR to the necrosis rate of the initial biopsy and thus increase its significance as previously applied by Vos et al. (13). Nevertheless, improvement of the current criteria for pathological response (residual viable tumor (RVT)) seems necessary as they were developed in the context of induction chemotherapy showing pathologic changes completely different to those of neoadjuvant immunotherapy (53). A promising approach seems to be the immune-related pathologic response criteria (irPRC) recently published by Cottrell et al. (53). According to the Authors, the observed discrepancy between CT imaging and pathological assessment of the residual tumor is explained by the area of immune-mediated tumor clearance referred to as regression bed. The regression bed is further characterized by tumor infiltrating lymphocytes with macrophages and tertiary lymphoid structures, tumor cell death or cholesterol clefts and tissue repair parameters, specifically neovascularization and proliferative fibrosis. To account for these changes, the irPRC are defined as irRVT = viable tumor area/total tumor bed area, whereby the total tumor bed area = regression bed + RVT + necrosis (53).

### New frontiers in neoadjuvant immunotherapy

Surgical therapy with curative intent is indicated for early stage HNSCC (7, 54, 55). In advanced cases, surgical tumor resection and neck dissection combined with radio(chemo)therapy are considered the most effective treatment options to date (5). **Cases A**, **B** and (39) outline the feasibility and potential effects of neoadjuvant ICI in recurrent or advanced HNSCC/OSCC prior to definitive surgical tumor treatment. ICI has not led to any delay in surgical procedures.

Shibata et al. describe a trend shift away from classical induction chemotherapy towards ICI for treatment of head and neck tumors (21). In case of immunotherapeutic approach, various explanatory models can be discussed for the significantly higher response rates in the neoadjuvant compared to the R/M setting (9–11). Response to ICI depends on local T cell infiltration (47, 56). As of now, most strategies focus on modulating tumor microenvironment in order to improve the overall efficacy of IT, not taking into consideration the possible role of tumor-draining lymph nodes and the immunomodulatory role of surgery prior to and after ICI. Since performing neck dissection results in higher rates of overall and disease-free survival (57) it plays a crucial role in surgical therapy of HNSCC. However, lack of lymphatic and potentially immunologically significant structures could have an impact on the efficacy of checkpoint inhibitors, thus explaining significantly higher response rates with neoadjuvant treatment prior to removal of lymphatic structures.

Two recent Phase III trials (MSLT-II and DeCOG-CLND; NCT00297895 and NCT02434107) demonstrated that completion lymph node dissection (CLND) for high risk melanoma treatment was not associated with better distant metastasis-free survival (DMFS) and OS outcomes (58, 59). In addition, the need for CLND to obtain better information on staging was disproved (60). In this regard, Eggermont, A.M.M. describes the paradigm shift from historical elective regional lymph node dissection (ELND) to sentinel lymph node dissection (SLND) plus CLND to sentinel lymph node dissection (SLND) alone in melanoma therapy as ground-breaking for the therapy of other entities, justified by a high response rate to immune checkpoint inhibition in melanoma (61). So far, the trend away from lymphadenectomy combined with ICI shifting to neoadjuvant is mainly limited to melanoma therapy (62). In other tumor entities accessible by ICI, such as HNSCC/OSCC, SLND as alternative for ELND remains controversial and should be further investigated in future trials. Recently, studies on murine HNSCC models by Saddawi-Konefka et al. showed that regional lymphablation eliminates the tumor response to ICI leading to worse overall survival (63). In addition, ELND led to repolarization of the tumor microenvironment and peripheral-immune compartments. The authors were able to map the murine cervical lymphatic system which allowed a precise modelling of lymphatic ablative therapy (ELND) (63). Animals were treated with different combinations of ELND and ICI without surgical treatment of the primary tumor site. Anti-CTLA4 or anti-PD-L1 treatment was administered 10 days after ELND or control skin incision. In this experimental layout, ELND counteracted the anti-PD-1 and anti-CTLA4 dependent reduction in primary tumor mass. Comparative analysis revealed a predominantly lymphocytic infiltrate and a less infiltrative cancer pattern in the primary tongue tumors of control animals compared to ELND animals under anti-CTLA4 ICI. Animals with ELND showed significantly increased CD45- cells, Myeloid derived suppressor cells (MDSCs) and M2-Type macrophages as well as a decrease in in CD8 and CD4 T-cell infiltration (63). These data support the potential use of ICI in a neoadjuvant setting with available lymphatic structures and motivate further investigation of SLND for cN0 HNSCC.

With regard to single-agent ICI, a recent meta-analysis by Masarwy et al. found no superiority of one particular anti-PD-1/PD-L1 agent over another (64). As of now, the addition of CTLA-4 antibodies (**Case F**) to standard anti PD-L1 ICI seems feasible without achieving significantly better results (64). The completed phase 2 study by Schoenfeld et al. investigated the administration of Nivolumab compared to Nivolumab and Ipilimumab (CTLA-4 antibody) and reported feasible use and promising response rates in the neoadjuvant setting for both cases. However, no significant difference was found between both study arms (38). Correspondingly, the IMCISION study showed that both Nivolumab alone and Nivolumab + Ipilimumab can be used successfully without significant differences in pathological response (65). Administering an additional tumor (immune-) microenvironment modulating drug, e.g. a tyrosine kinase inhibitor, could increase ICI efficacy. Oliva et al. reported a profound clinical and pathological response to preoperative Sitravatinib and Nivolumab, although the restricted patient number and lack of single-agent arms limit the study and necessitate further investigation (14). There is promising potential for drugs targeting the tumor microenvironment as immunological preconditioning in combination with classical immune checkpoint inhibition and should therefore be further explored.

Regarding the association of HPV and HNSCC, recent studies suggest that HPV-positive tumors of the oropharynx tumors respond significantly better to immunotherapy than HPV-negative ones (66, 67). In addition, the Keynote-012 and Checkmate-141 trials indicate a better response to ICI in HPV-positive tumors (9, 68). With the presence of functional HPV-specific PD-1+ TCF-1+ CD45RO+ stem-like CD8 T cells with proliferative capacity, Eberhardt et al. recently demonstrated that the cellular machinery to respond to PD-1 blockade exists in HPV-positive head and neck cancer (69). Nevertheless, HNSCC should be considered as a heterogeneous group of tumors. As Hübbers C.U. and Akgül B. noted, in contrast to the proven prognostic relevance of biologically active HPV infection in squamous cell carcinoma of the oropharynx, no such definite association was found for OSCC (70). The decision-making in case of the presented patients was not influenced by this due to a consistently negative HPV status.

Given these rapidly changing and exciting new frontiers, where does surgery situate itself? Is salvage surgery a viable option for patients in a palliative situation after failure of ICI or at stable disease state under immunotherapeutic treatment?

### Salvage surgery and immunotherapy

The clinical course of **Cases E and F** shows that salvage surgery can be performed successfully after ICI failure and can lead to an improvement in clinical condition. Since studies on salvage surgery in stable disease states under ICI and its effect on quality of life (QOL) are currently lacking, **Cases C and D** demonstrate the clinical feasibility and possible benefits.

In view of the current study landscape, salvage surgery for locoregional recurrence is considered controversial. According to Horn et al, who prospectively studied Salvage surgery with microvascular reconstruction for recurrent OSCC, it is a viable treatment option with acceptable morbidity and high success rate (34, 35). Similar results were reported by Patil et al. for patients with resectable locoregional recurrence without the option of curative re-irradiation (71, 72). Hamoir et al. and Saba et al. are more critical of salvage surgery due to high risk of recurrence at 30-40% and the significant risk of complications which is up to 67% (73, 74). All authors emphasise the urgency of new studies regarding the potentially synergistic effects of ICI and salvage surgery. Our **Cases C, D, E and F** demonstrate the feasibility and the improvement of clinical conditions through salvage surgery with limited complications even with complex surgical procedures requiring up to two microvascular flaps. If patients are ineligible for surgical therapy, all other treatment options should be considered. As stated by Altay-Langguth et al., radioimmunotherapy (re-irradiation with Nivolumab) might offer an effective treatment option in pre-irradiated patients (75).

Surgical debulking, whose effects on tumor progression have been controversial so far, could have alternative effects under immunotherapeutic treatment (76–78). Patients who opt for salvage surgery have most likely already undergone surgical resection of the primary tumor, including neck dissection. This procedure may have significant impact on the efficacy of checkpoint inhibitors as discussed earlier. In contrast to the potential negative impact of surgical lymphadenectomy on systemic ICI success, reduction of tumor mass in case of recurrence could play an important role in the efficacy of ICI in heavily pre-treated patients. Oppel et al. describe the emergence of ICI resistant tumor cell clones as a similar mechanism to classical chemotherapy resistance based on additionally acquired genetic alterations (76, 79). To avoid this, it is proposed to reduce genetic heterogeneity prior to molecular targeting, which could reduce the statistical probability of tumor recurrence triggered by resistant clones. Tumor debulking is suggested as a non-specific remedy for this, as currently successfully applied in clinical treatment of ovarian cancer (76). Additionally, initial experimental studies describe surgical debulking as a promoter for macrophage recruitment and therefore as a potential trigger for tumor phagocytosis in combination with ICI (77). However, increased macrophage infiltration in OSCC in the absence of ICI could also contribute to tumor progression (80, 81). Khong et al. observed a similar immunomodulatory effect of incomplete tumor debulking alone and investigated the proportion of CD4+ and CD8+ T cells in the postoperative tumor microenvironment of mice with malignant mesothelioma. Further research demonstrated that this postoperative tumor microenvironment is amenable to immunomodulatory drugs such as TLR7 agonists and anti-CD40, resulting in improved outcomes and tumor regression in 25% of cases (78).

Regarding recurrent HNSCC in particular Ritter at al. describe the feasibility of function preserving tumor debulking combined with brachytherapy and a simultaneous Cetuximab-Paclitaxel protocol as second-line treatment. Adding the Cetuximab-Paclitaxel protocol to standard tumor debulking and brachytherapy resulted in significantly higher disease-free survival and overall survival (82). Yet, the specific relationship between tumor debulking and additional systemic therapy remains unclear.

As **Case D** demonstrates, salvage surgery led to a significant improvement in quality of life and disease-free survival. The current tumor follow-up showed no signs of recurrence. Although this case cannot be considered a direct case of tumor debulking due to its R0 situation, the present field cancerization allows a similar conclusion. In addition, **Cases C, E and F** demonstrate the potentially life-prolonging and quality of life improving effect of salvage surgery in the context of immunotherapeutic treatment in selected cases.

### Conclusion

ICI is rapidly expanding its frontiers into the neoadjuvant setting. However, the benefits of neoadjuvant ICI over conventional therapy of advanced head and neck cancer with surgical resection and adjuvant RCT has not yet been demonstrated in prospective phase 3 trials. Phase 2 studies with small patient collectives show that neoadjuvant ICI could improve the prognosis of advanced head and neck cancer. Yet, timing and surrogate markers for response remain unclear. For neoadjuvant ICI monitoring regarding pathological tumor response or tumor necrosis rate, we suggest correlation between the initial biopsy and the definite tumor resectate in order to increase its significance as a surrogate marker. Scheduling of neoadjuvant ICI should be further investigated, as recent studies indicate better outcomes with shorter time frames.

The currant multimodal treatment including immunotherapy after failure of primary surgery and RCT frequently confronts the surgeon with heavily pretreated patients and tumor progression. Salvage surgery remains a feasible therapy concept despite an increasing amount of systemic second- and third-line treatment options. Decision-making on surgical intervention in case of a salvage surgery remains, to this day, an individual choice.

## Data availability statement

The original contributions presented in the study are included in the article/Supplementary Material. Further inquiries can be directed to the corresponding author.

## Ethics statement

The studies involving human participants were reviewed and approved by Ethics Committee of the Friedrich-Alexander University Erlangen-Nuremberg. Application number: 22-183-Br. The patients/participants provided their written informed consent to participate in this study. Written informed consent was obtained from the individual(s) for the publication of any potentially identifiable images or data included in this article.

## Author contributions

MW, MO, MK, RL, C-PN, TM, MH, A-OG, MiE, YF, T-OB, and JG participated in the examination and treatment of one or more of the patients and collected the data. MaE participated in the pathological diagnosis. MO and MW discussed the case and data. MO and MW wrote the manuscript. JR, RL, and T-OB aided with manuscript writing and language prettification. JR significantly contributed to the figure design. All authors read and approved the final manuscript.

## Conflict of interest

MW has consulting contracts with the pharmaceutical companies Sanofi and Bristol Myers Squibb. MiE participated in an advisory board by Bristol Myers Squibb.

The remaining authors declare that the research was conducted in the absence of any commercial or financial relationships that could be construed as a potential conflict of interest.

## Publisher’s note

All claims expressed in this article are solely those of the authors and do not necessarily represent those of their affiliated organizations, or those of the publisher, the editors and the reviewers. Any product that may be evaluated in this article, or claim that may be made by its manufacturer, is not guaranteed or endorsed by the publisher.

## Abbreviations

HNSCC, Head and neck squamous cell carcinoma; IT, Immunotherapy; R/M, recurrent or metastatic; CSCC, Cutaneous squamous cell carcinoma; NCCN, National Comprehensive Cancer Network; cPR, Complete pathologic response; MPR, Major pathologic response; CT, Computed tomography; OSCC, Oral squamous cell carcinoma; TPS, Tumor proportion score; IC-Score, Inflammatory cell score; CPS, Combined positive score; pTR, Pathologic tumor response; RCT, Radiochemotherapy; RVT, residual viable tumor; ICI, Immune checkpoint inhibition; irPRC, Immune-related pathologic response criteria; MDSCs, Myeloid derived suppressor cells; TCF-1, T cell factor-1.
